# THE HEALTH OF MALE VETERANS IN LATER LIFE

**DOI:** 10.1093/geroni/igz038.783

**Published:** 2019-11-08

**Authors:** Janet M Wilmoth, Scott D Landes, Andrew S London

**Affiliations:** Syracuse University, Syracuse, New York, United States

## Abstract

Veterans have the opportunity to accrue health-promoting “military capital,” but they are also at risk of experiencing a “military hazard” effect that undermines later-life health and mortality outcomes. Given these possibly competing effects, there is substantial heterogeneity in physical and mental health among older male veterans. The health and mortality outcomes of older veterans who were not substantially harmed during military service appear to be just as good as, if not better than, those of nonveterans. However, older veterans who served in-theater, were exposed to combat or hazardous chemicals, and/or were physically or psychologically harmed during service tend to have worse health and higher mortality than non¬veterans. Some older veterans with these experiences struggle with life-long or late-onset PTSD, while others exhibit resilience and posttraumatic growth. Additional population-level, life-course research is needed on specific war-era cohorts to identify the mechanisms that shape veteran status differences in late-life health and mortality.

